# APPLYING A TRAUMA-INFORMED DEI PRACTICE MODEL TO TRANSFORM CARE CULTURE

**DOI:** 10.1093/geroni/igad104.2389

**Published:** 2023-12-21

**Authors:** Jennifer Morgan, Kendra Jason, Leigh-Anne Royster

**Affiliations:** Georgia State University, Atlanta, Georgia, United States; University of North Carolina at Charlotte, Charlotte, North Carolina, United States; Duke, Durham, North Carolina, United States

## Abstract

Collective trauma is a psychological distress experienced by large numbers of people or communities in response to shared trauma. It has been documented that the traumatic impacts of COVID-19 in concert with discrimination based on any marginalized identity (e.g., racialized, gender, and sexual minorities; poor, chronically-ill) leads to poorer health, work, and life outcomes. Marginalized older adults who have experienced discrimination over the life course are at increased risks for these challenges as they are more likely than any other group to experience infection, hospitalization, or death due to COVID-19. For older workers, they are more likely to remain unemployed or face more challenges at work than younger workers. Additionally, long-term care services and supports serving marginalized populations are more likely to have staff that have marginalized identities themselves and deal with similar challenges. We offer a conceptual model designed to transform care culture in ways that explicitly foster diversity, equity, inclusion and ultimately belonging and empowerment of individual community members. This conceptual model is guided by three founding principles: DEI (diversity, equity and inclusion), intersectionality, and trauma-informed care. This approach allows for care practitioners to address the intersecting structural and compounding challenges of COVID-19 and discrimination, instead of treating them as separate non-competing factors. By mapping out the evidence of collective trauma experienced by many minoritized and older workers; and those in aging services and long-term care sectors, we explain the principles of a trauma-informed approach and how retention and empowerment practices can support transformation of care culture.

